# P-347. Investigating Risk Factors for *Candida auris* Bloodstream Infections: A Case-Control Study from Los Angeles County

**DOI:** 10.1093/ofid/ofae631.549

**Published:** 2025-01-29

**Authors:** Jennifer Nguyen, Kelsey OYong, Zachary A Rubin

**Affiliations:** Los Angeles County Department of Public Health, Los Angeles, California; Los Angeles County Department of Public Health, Los Angeles, California; Los Angeles County Department of Public Health, Los Angeles, California

## Abstract

**Background:**

*Candida auris* (*C. auris*) has emerged as a significant pathogen due to its ability to cause bloodstream infections (BSIs) in highly vulnerable patient populations. Understanding the risk factors associated with *C. auris* BSIs is crucial for implementing targeted interventions to mitigate the burden of infection and ensure patient safety. In Los Angeles County (LAC), all *C. auris* cases are reportable to the Department of Public Health (DPH).

**Methods:**

We conducted a case-control study to identify risk factors associated with *C. auris* BSIs. Using data reported to LAC from June 2019 to April 2024, cases included persons initially colonized with *C. auris* who subsequently developed confirmed *C. auris* BSI, while controls consisted of persons colonized with *C. auris* without *C. auris* identified from a clinical specimen site. *C. auris* cases and controls were identified via required laboratory and provider reporting of all positive *C. auris* tests. Data on various risk factors were collected from provider case reports that were completed on all *C. auris*-positive persons, excluding cases with missing risk factor data. We used logistic regression to estimate the adjusted odds ratios of a colonized case acquiring a *C. auris* BSI compared to only colonized persons, adjusting for age, sex, race/ethnicity, and healthcare facility where the positive specimen was collected.

**Results:**

Of the 174 cases and 2,602 controls identified, increased odds of BSI were observed among patients with prior antifungal use (OR: 3.58, 95% CI: 1.12-11.41) and hemodialysis (OR: 2.33, 95% CI: 1.05-5.16). Other risk factors such as ventilator use, tracheostomy, central venous catheter presence, and wound presence showed no statistically significant associations.

**Conclusion:**

Targeted infection prevention strategies are vital to mitigate *C. auris* BSI burden. Rigorous aseptic techniques during hemodialysis and judicious use of antifungal agents may reduce *C. auris* BSI risk. Although adjusted for in this analysis, specialized attention to infection control in healthcare facilities such as long-term acute care hospitals may be warranted, given their unique patient populations and care settings. Further investigation is warranted to identify additional strategies to mitigate *C. auris* BSI risk.

**Disclosures:**

**All Authors**: No reported disclosures

